# A Universal Approach to Eliminate Antigenic Properties of Alpha-Gliadin Peptides in Celiac Disease

**DOI:** 10.1371/journal.pone.0015637

**Published:** 2010-12-16

**Authors:** Cristina Mitea, Elma M. J. Salentijn, Peter van Veelen, Svetlana V. Goryunova, Ingrid M. van der Meer, Hetty C. van den Broeck, Jorge R. Mujico, Veronica Monserrat, Luud J. W. J. Gilissen, Jan Wouter Drijfhout, Liesbeth Dekking, Frits Koning, Marinus J. M. Smulders

**Affiliations:** 1 Department of Immunohematology and Blood Transfusion, Leiden University Medical Center, Leiden, The Netherlands; 2 Plant Research International, Wageningen UR, Wageningen, The Netherlands; 3 Allergy Centre Wageningen, Wageningen, The Netherlands; University of South Florida College of Medicine, United States of America

## Abstract

Celiac disease is caused by an uncontrolled immune response to gluten, a heterogeneous mixture of wheat storage proteins, including the α-gliadins. It has been shown that α-gliadins harbor several major epitopes involved in the disease pathogenesis. A major step towards elimination of gluten toxicity for celiac disease patients would thus be the elimination of such epitopes from α-gliadins. We have analyzed over 3,000 expressed α-gliadin sequences from 11 bread wheat cultivars to determine whether they encode for peptides potentially involved in celiac disease. All identified epitope variants were synthesized as peptides and tested for binding to the disease-associated HLA-DQ2 and HLA-DQ8 molecules and for recognition by patient-derived α-gliadin specific T cell clones. Several specific naturally occurring amino acid substitutions were identified for each of the α-gliadin derived peptides involved in celiac disease that eliminate the antigenic properties of the epitope variants. Finally, we provide proof of principle at the peptide level that through the systematic introduction of such naturally occurring variations α-gliadins genes can be generated that no longer encode antigenic peptides. This forms a crucial step in the development of strategies to modify gluten genes in wheat so that it becomes safe for celiac disease patients. It also provides the information to design and introduce safe gluten genes in other cereals, which would exhibit improved quality while remaining safe for consumption by celiac disease patients.

## Introduction

Celiac Disease (CD) is an intestinal T cell-mediated disease caused by the gluten fraction of wheat or the homologous proteins from barley or rye. CD has prevalence between 0.5 and 2% in human populations [1] and is characterized by a chronic intestinal inflammation upon ingestion of gluten proteins. Recently, the molecular aspects have been comprehensively addressed in several review papers [2]–[5]. In short, in CD patients CD4+ T cells are present in the lamina propria that secrete interferon-gamma upon recognition of gluten-derived peptides bound to HLA-DQ2 or HLA-DQ8 molecules present on antigen presenting cells. Strikingly, most of the gluten peptides implicated in CD require modification by the enzyme tissue transglutaminase before they can bind to the disease-predisposing HLA-DQ molecules and trigger T cell responses [2]–[5]. In addition to the adaptive CD4+ T cell response to gluten, CD is characterized by the upregulation of IL-15, an intraepithelial T cell infiltrate expressing the NKG2D receptor, and the overexpression of a ligand for NKG2D (MICA) [6], [7].

Many gluten peptides with T cell stimulatory properties have now been identified. Such peptides have been found in wheat α-, γ-, and ω-gliadins as well as in low molecular weight (LMW) and high molecular weight (HMW) glutenins [8]–[14]. Several studies have demonstrated that peptides derived from α-gliadins induce strong T cell responses in the large majority of patients, while responses to the other peptides are less frequently found [8]–[10], [13]. An α-gliadin derived 33-mer peptide (amino acid sequence LQLQPFPQPQLPYPQPQLPYPQPQLPYPQPQPF) was identified that encodes six partially overlapping T cell epitopes and has very potent T cell stimulatory properties [13]. It harbours the p56–75 peptide (LQLQPFPQPQLPYPQPQLPY) that has been identified as the dominant gluten epitope [9], [10]. Furthermore, α-gliadins are the only gluten molecules that harbor the p31-43/49 peptide that has been implicated in the innate immune response induced by gluten [7].

The α-gliadins are a gene family encoded by the *Gli-2* loci, *Gli-A2, Gli-B2 and Gli-D2,* located on the short arm of three homoeologous chromosomes (6AS, 6BS and 6DS) of hexaploid bread wheat (*Triticum aestivum L.*). These loci may contain from 25–35 to even 150 α-gliadin genes per haploid genome [15]–[17], although most of these (72–95%) are presumably pseudogenes [16], [17]. Sequencing of genomic α-gliadin clones from hexaploid bread wheat enabled to differentiate the sequences according to their loci *Gli-A2, Gli-B2* and *Gli-D2* based on genome-specific SNPs [16], [17]. Relevant for CD, the occurrence and frequency of the HLA-DQ2 epitopes DQ2-Glia-α1, DQ2-Glia-α2 and DQ2-Glia-α3 (previously designated Glia-α20, ref. 12] and the HLA-DQ8 T-cell epitope DQ8-Glia-α1 also differs between the loci [17]. This was corroborated by the observation that T cell clones specific for the DQ2-Glia-α2 epitope did not recognise gluten derived from diploid species carrying the S-genome, ancestrally related to the B genome of bread wheat, while gluten derived from diploid A- and D-genome species was recognized [18]. Variation in T cell stimulatory capacity of cereal-derived gluten was observed with other T cell clones as well [19]–[21]. Indeed, differences have been observed in the T cell stimulatory capacity of pasta and bread wheat varieties [22], [23], but none were safe for CD patients.

Given the overall importance of the α-gliadins in CD we set out to determine the naturally existing sequence variation in CD epitopes as deduced from α-gliadin transcripts from developing wheat grains. The immunogenic potential of these epitope variants was subsequently tested in T-cell proliferation assays. This produced insight in which key amino acid changes are sufficient to abolish T-cell recognition. Finally, we verified that the observed differences in antigenicity of α-gliadin peptides derived from diploid species corresponded to differences in the antigenicity of the gluten from these species, ancestrally related to bread wheat. Altogether the results offer a molecular basis for differential CD toxicity of the wheat genomes. Based upon these results we present a rational strategy to develop genes that encode α-gliadins that are safe for consumption by celiac disease patients.

## Results

### Genetic variation in α-gliadin (*Gli-2*) transcripts

In total 3022 expressed α-gliadin sequences (expressed sequence tags and mRNA sequences from NCBI and Unigene) originating from 11 different *T. aestivum* L. varieties were analyzed. These α-gliadin transcripts were grouped into 55 contigs with at least 90% sequence homology. Forty per cent of the α-gliadin transcripts clustered with A genomic sequences and were attributed to locus *Gli-A2*, 35% originated from *Gli-D2* and only 25% came from *Gli-B2* (**Fig. S1**). After tracing all non-synonymous DNA polymorphisms 83 different transcript contigs were obtained for the 3′ region of the gene that each contained at least four sequence equivalents. This indicates a high sequence diversity among expressed α-gliadin sequences in these 11 *T. aestivum* L. varieties.

### Variants of T cell stimulatory and innate stimulatory sequences

The N-terminal region of α-gliadins contains the p31-43 epitope implicated in the innate immune response, and the immunodominant DQ2-Glia-α1 and DQ2-Glia-α2 epitopes as well as the DQ2-Glia-α3 T cell epitope. The carboxyl-terminal part encodes the immunodominant DQ8-Glia-α1 T cell epitope (**Table 1**). To obtain information on the immunogenic potential of the various α-gliadins, the translated amino acid sequences for each locus were checked for the presence of canonical T cell epitopes and variants thereof. **Tables 2** **,** **3** **,** **4** **,** **5** present the most frequently expressed epitope variants (>5 transcripts each).

**Table 1:** Amino acid sequences of α-gliadin derived peptides.

	Immune response	Restriction element	Core-Sequence
DQ2-Glia-α1	Adaptive	HLA-DQ2	P{F/Y}PQPQLPY
DQ2-Glia-α2	Adaptive	HLA-DQ2	PQPQLPYPQ
DQ2-Glia- α3	Adaptive	HLA-DQ2	FRPQQPYPQ
DQ8-Glia-α1	Adaptive	HLA-DQ8	QGSFQPSQQ
P31-43	Innate	not applicable	PGQQQPFPPQQPY

Five antigenic peptides derived from α-gliadins are known to be involved in celiac decease. For the peptides that can provoke an adaptive immune response in CD patients (HLA-DQ2 restricted or HLA-DQ8 restricted) the minimal 9-mer “canonical” epitope cores are shown. One peptide, **p31-43,** is known to be involved in an innate immune response observed in CD. For each of the epitopes is specified the name, which immune response it evokes, the restriction element and the amino acid sequence.

**Table 2:** HLA-DQ2-Glia-α1 epitope variants expressed in bread wheat.

	*Gli-A2*	*Gli-B2*	*Gli-D2*	N
PFPQPQLPY	466		520	986
P**Y**PQPQLPY			93	93
PF**L**QPQLPY	106			106
PF**S**QPQLPY	94			94
PFPQPQL**S**Y	7		54	61
PFP**H**PQLPY			29	29
PFPQ**A**QLPY			6	6
Total	673		702	1375

Expressed variants of the DQ2-Glia-α1 epitope represented by ≥5 transcripts and the number of transcripts per epitope variants per chromosomal locus (*Gli-A2*, *Gli-B2* or *Gli-D2*). N =  total transcript count per variant. In bold: amino acid variation.

**Table 3:** DQ2-Glia-α2 epitope variants expressed in bread wheat.

	*Gli-A2*	*Gli-B2*	*Gli-D2*	N
PQPQLPYPQ			607	607
PQPQLPY**S**Q	431			431
**FP**PQLPYPQ		382		382
**L**QPQLPY**S**Q	106			106
**FL**PQLPYPQ		31		31
**S**QPQLPY**S**Q	85			85
PQPQL**S**YPQ			54	54
PQP**H**LPYPQ			30	30
P**H**PQLPYPQ			29	29
PQPQL**S**Y**S**Q	7			7
PQ**A**QLPY**S**Q			6	6
PQPQ**PQ**YPQ		6		6
Total	629	419	726	1774

Expressed variants of the DQ2-Glia-α2 epitope represented by ≥5 transcripts and the number of transcripts per epitope variants per chromosomal locus (*Gli-A2*, *Gli-B2* or *Gli-D2*). N =  total transcript count per variant. In bold: amino acid variation.

**Table 4:** DQ2-Glia-α3 epitope variants expressed in bread wheat.

	*Gli-A2*	*Gli-B2*	*Gli-D2*	N
FRPQQPYPQ	650		449	1099
*F****P****PQQPYPQ*	732	687	606	2025
*F****PS****QQPYPQ*		179	8	187
FRPQQ**S**YPQ			157	157
F**P**PQQPYP**H**		148		148
F**PA**QQPYPQ		56		56
F**P**PQQ**S**YPQ		18		18
F**PG**QQPYPQ		20		20
F**Q**PQQPYPQ	13			13
F**L**PQQPYPQ			8	8
FRPQQ**Q**YPQ			6	6
Total	1395	1108	1234	3737

Expressed variants of the DQ2-Glia-α3 epitope represented by ≥5 transcripts and the number of transcripts per epitope variants per chromosomal locus (*Gli-A2*, *Gli-B2* or *Gli-D2*). *In italics*: DQ2-Glia-α3 variants located on the position of the innate responsive element, p31-43. N =  total transcript count per variant. In bold: amino acid variation.

**Table 5:** DQ8-Glia-α1 epitope variants expressed in bread wheat.

	*Gli-A2*	*Gli-B2*	*Gli-D2*	N
QGSFQPSQQN		186	381	567
QGSF**R**PSQQN	409			409
QG**F**FQPSQQN			114	114
QGSF**R**P**F**QQN	103			103
Q**V**SFQPSQ**L**N		112		112
QGSFQ**S**SQQN		111		111
QGSFQP**F**QQN		4	27	31
QG**F**FQP**F**QQN			6	6
Total	512	413	528	1453

Expressed variants of the DQ8-Glia-α1 epitope, represented by ≥5 transcripts and the number of transcripts per epitope variants per chromosomal locus (*Gli-A2*, *Gli-B2* or *Gli-D2*). N =  total transcript count per variant. In bold: amino acid variation.

We observed that canonical DQ2-Glia-α1 (**Table 2**) and DQ2-Glia-α3 epitopes (**Table 4**) were only present in *Gli-A2* and *Gli-D2* transcripts and that the canonical DQ2-Glia-α2 motif (**Table 3**) was unique for *Gli-D2* transcripts (72% of all *Gli-D2* transcripts contained at least one DQ2-Glia-α2 epitope core). DQ8-Glia-α1 canonical epitopes were present in *Gli-D2* and *Gli-B2* transcripts only (**Table 5**). The canonical p31-43 motif was not restricted to transcripts from a specific locus and was found in transcripts from all α-gliadin loci.

In addition to the canonical epitope motifs, a large series of sequence variants with one or two amino acid substitutions were detected (**Tables 2** **,** **3** **,** **4** **,** **5**). The large majority of the *Gli-B2* gliadins contained sequence variants of the DQ2-Glia-α1, DQ2-Glia-α2, and DQ2-Glia-α3 epitopes with two amino acid substitutions. Furthermore, *Gli-A2* transcripts harbored a proline to serine substitution at position 8 (p8) of the DQ2-Glia-α2 epitope and a sequence variant (QGSF**R**P(S/F)QQN, amino acid substitution in bold) of the DQ8-Glia-α1 epitopes. Importantly, the 33-mer peptide (LQLQPFPQPQLPYPQPQLPYPQPQLPYPQPQPF) that is highly resistant to degradation in the gastrointestinal tract and contains six overlapping DQ2-Glia-α1 and DQ2-Glia-α2 epitopes, conferring superior T cell stimulatory properties [13], was only observed in a subset of the α-gliadins from the *Gli-D2* locus and never in the α-gliadins from the *Gli-A2* and *Gli-B2* loci. The latter expressed substantially truncated versions of the 33-mer (**Fig. S2**).

### T cell stimulatory capacity of α-gliadin derived peptides

Several of the amino acid variants that we found in the α-gliadin transcriptome have never been described before while some have been described but were never tested for their T cell stimulatory capacity. In order to determine which variants are capable of inducing T cell responses, the DQ2-Glia-α1, DQ2-Glia-α2, DQ2-Glia-α3, and DQ8-Glia-α1 variants were synthesized as 15- or 16-mer peptides and tested for their capacity to bind to HLA-DQ2 and induce *in vitro* proliferation of HLA DQ2- or DQ8-restricted T cell clones.

#### DQ2-Glia-α1 variants

Virtually all peptides that carry the canonical 9-mer Glia-α1 epitope core P_1_{F/Y}_2_P_3_Q_4_P_5_E_6_L_7_P_8_Y_9_ (in which the glutamic acid at p6 is introduced by TG2-deamidation of the original glutamine) were able to stimulate DQ2-Glia-α1 T cells. Only an arginine residue at the position preceding the epitope core diminished T cell stimulation. In the core sequence, several amino acid substitutions diminished or abolished the T cell stimulatory capacity, such as a proline to serine substitution at p3 or p8 and a proline to alanine substitution at p5 (**Table 6**, no. 3). Peptides in which an amino acid was deleted at p3 or p4 were not causing any proliferation of the T cells. Strikingly, such safe peptides are all from locus *Gli-B2* (**Table 6** **, Fig. S3**).

**Table 6:** T cell proliferation and HLA-DQ2 binding capacity of DQ2-Glia-α variants.

No.	Peptide	locus	IC50DQ2-Glia-α1	Glia-α1T cell clone	IC50DQ2-Glia-α2	Glia-α2T cell clone
1	QLQPFPQPELPYPQP**E**	*Gli-D2*	5	+	18	+
2	QLQPFPQPELPYPQPQ	*Gli-D2*	5	+	34	+
3	QLQPFPQ**A**ELPY**S**QPQ	*Gli-D2*	8	±	11	-
4	QLQPFPQPEL**S**YPQPQ	*Gli-D2*	12	-	24	-
5	QLQ**R**PFPQPELPYPQP**E**	*Gli-D2*	14	±	19	+
6	QLQPFPQPELPY**TH**	*Gli-D2*	21	+	21	-
7	QLQPFP**H**PELPYPQPQ	*Gli-D2*	42	+	41	±
8	QLQPF**S**QPELPY**S**QPQ	*Gli-A2*	7	±	32	-
9	QLQPFPQPELPY**S**QPQ	*Gli-A2*	57	+	41	-
10	QLQPFPQPELPY**S**QPE	*Gli-A2*	67	+	24	-
11	QPQPF**L-**PELPYPQPE	*Gli-B2*	4	-	4	±
12	QPQPF-QPELPYPQPE	*Gli-B2*	8	-	8	+
13	QPQ**E**FP-PELPYPQPE	*Gli-B2*	45	-	45	-
14	QPQ**Q**FP-PELPYPQPE	*Gli-B2*	86	-	86	-
15	QPQPFP-PELPYPQ**T**QP	*Gli-B2*	nd	-	nd	-
			**IC50** **DQ2-Glia-α3**	**Glia-α3** **T cell clone**		
16	QPFRPEQPYPQPQPQ	*Gli-A2/D2*	35	+		
17	QPF**P**PEQPYPQPQPQ	*Gli-A2/D2/B2*	3080	-		
			**IC50** **DQ8-Glia-α1**	**DQ8-Glia-α1** **T cell clone**		
18	LGEGSFQPSQENP	*Gli-D2/B2*	16	+		
19	LGEGSF**R**PSQENP	*Gli-A2*	33	-		
20	LGEG**F**FQPSQENP	*Gli-D2*	nd	-		

Variants of the DQ2-Glia-α1 and DQ2-Glia-α2 epitopes as encoded by the α-gliadin transcriptome were synthesized as deamidated 14- to 17-mer peptides (column 1, underlined: DQ2-Glia-α1/α2 epitope region) and tested for stimulation of DQ2-Glia-α1 and DQ2-Glia-α2 specific T cell clones in a proliferation assay. ± = 100 times reduced T cell stimulation compared to the ‘canonical’ epitope; - = 1000 times reduced T cell stimulation compared to the ‘canonical’ epitope. For each epitope shorter versions of the peptide variant, including the putative epitope core flanked by at least two amino acids, were tested in a cell free *in vitro* peptide binding assay for binding to HLA-DQ2 antigen presenting cells (lysates from HLA-DR3/DQ2 positive EBV-transfored B-cells). IC50 DQ2-Glia-α1/α2 = mean value of the half maximal inhibitory concentration (IC_50_) returned by the binding assays for respectively DQ2-Glia-α1 and DQ2-Glia-α2 epitope variants. IC_50_ values were calculated based on the observed competition between the tested peptides and biotin-labelled indicator peptides and indicate the concentration of the tested peptide required for half maximal inhibition of the binding of the indicator peptide.

#### DQ2-Glia-α2 variants

Full responses of DQ2-Glia-α2 T cells were only observed against peptides that carry the core P/F_1_Q_2_P_3_E_4_L_5_P_6_Y_7_P_8_Q_9_. A deletion of the glutamine at p2, indicative for α-gliadins from locus *Gli-B2,* or substitution of this glutamine by histidine diminished or abolished the stimulatory capacity. Furthermore, a single substitution of the proline for an serine residue at either p6 or p8 abolished the T cell stimulating capacity. The latter substitution is found in α-gliadins from locus *Gli-A2* (**Table 6**).

#### DQ2-Glia-α3 variants

Also for this epitope several amino acid substitutions were found to destroy T cell stimulatory capacity, including an arginine to proline substitution at p2, which is found in α-gliadins from *Gli-B2* (**Table 6**).

#### DQ8-Glia-α1 variants

While several amino acid substitutions were found to influence T cell recognition of the canonical sequence Q_1_G_2_S_3_F_4_Q_5_P_6_S_7_Q_8_Q_9_, a single serine to phenylalanine substitution at p3 and a single glutamine to arginine substitution at p5 were found to completely destroy T cell stimulatory properties (**Table 6**). While the former T cell stimulatory variants are found in α-gliadins from *Gli-D2* and *Gli-B2*, the glutamine to arginine variants are from *Gli-A2* (**Table 5** **, Fig. S2**).

Thus, the α-gliadins encoded by *Gli-A2* of bread wheat are marked with a specific single amino acid substitution (P to S at p8) and lack the capacity to stimulate Glia-α2 T cell clones, whereas α-gliadins encoded by *Gli-B2* carry a deletion that prevents Glia-α1 T cell clone stimulation. Alpha-gliadins with the two intact epitopes, Glia-α1 and Glia-α2, are encoded by *Gli-D2* (**Tables 2** **and** **3**).

### T cell stimulatory capacity of diploid wheat accessions

Previously, differential reactivity of α-gliadin specific T cell clones against gluten extracts from diploid wheat accessions has been reported [18], [19]. In order to link such differential reactivity to the presence of specific epitope variants, a panel of diploid wheat accessions containing either the A, S or D genome was tested for their reactivity of an α-gliadin specific monoclonal antibody (mAb) and T cells.

**Figure 1:**
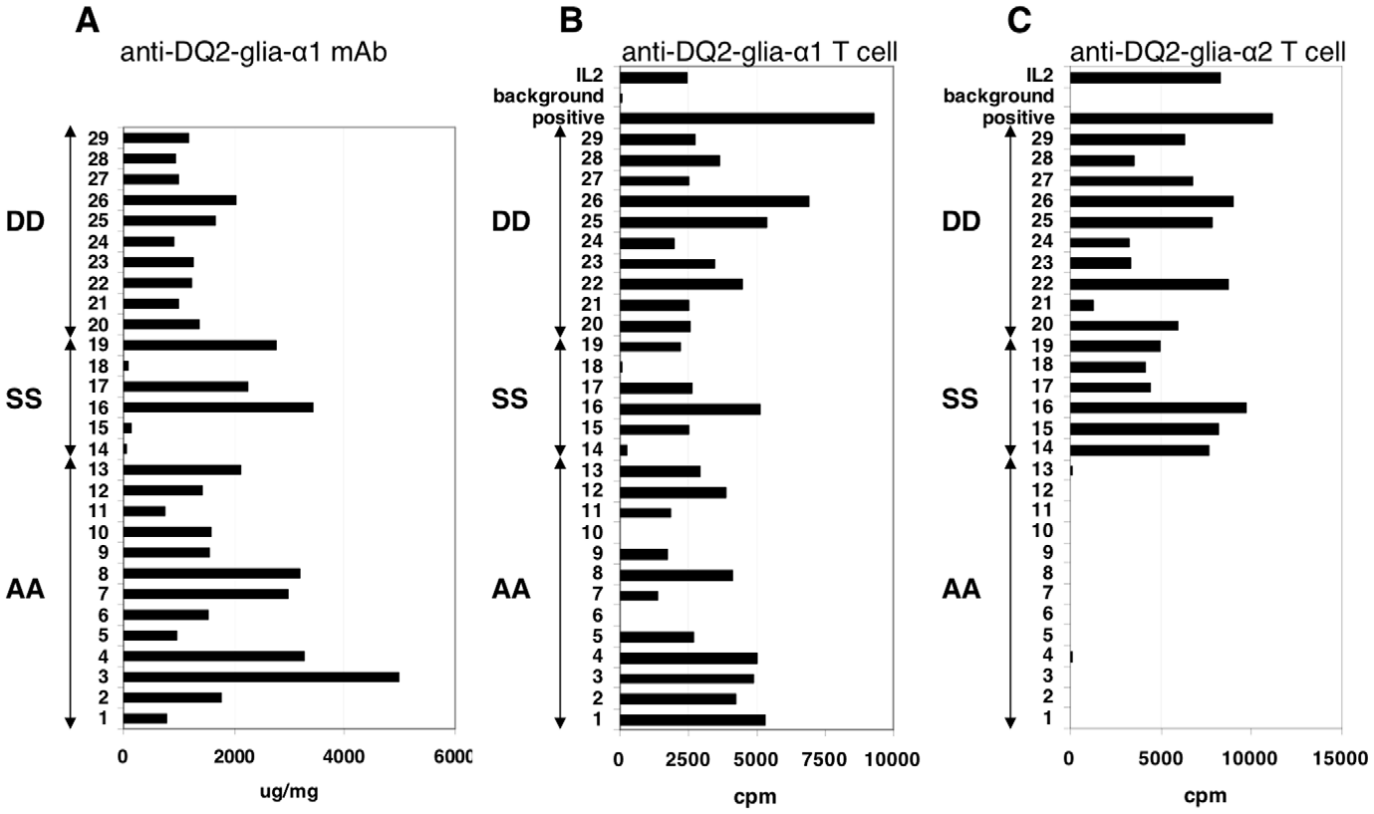
Presence of DQ2-Glia-α1 and DQ2-Glia-α2 epitopes in diploid wheat. Pepsin-trypsin digests of 29 diploid wheat accessions were prepared and tested in a competition ELISA with a mAb specific for a sequence partially overlapping with the DQ2-Glia-α1 and DQ2-Glia-α2 epitopes and after deamidation with T cell clones specific for the DQ2-Glia-α1 and DQ2-Glia-α2 epitopes. A: results of the competiton ELISA. B: T cell proliferation assay with a DQ2-Glia-α1 specific T cell clone. C: T cell proliferation assay with a DQ2-Glia-α2 specific T cell clone. IL-2: proliferation of the T cell clone under the influence of interleukin-2. Background: proliferation of the T cells in the presence of antigen presenting cells but no antigen. Positive control: proliferation of the T cell clone in the presence of a synthetic peptide encoding the specific α-gliadin epitope and antigen presenting cells. cpm: counts per minute. AA: diploid accessions with an A genome; SS: diploid accessions with an S genome; DD: diploid accessions with a D genome.

Pepsin-trypsin digests of gluten extracts from kernels of 29 diploid *Triticum* and *Aegilops* accessions were prepared and tested in a competition ELISA with a mAb specific for a sequence partially overlapping with the DQ2-Glia-α1 and DQ2-Glia-α2 epitopes **(** **Fig. 1A** **)** and, after treatment with TG2, with T cell clones specific for either the DQ2-Glia-α1 and DQ2-Glia-α2 epitope (**Fig. 1B and 1C**). The results indicate that the mAb reacts strongly with all extracts except three derived from diploids expressing the S genome (**Fig. 1A**). Similarly, and in agreement with previous results [18], extracts from A, S and D origin were capable of stimulating the DQ2-Glia-α1 specific T cell clone (**Fig. 1B**) while the extracts of the diploids expressing the A genome failed to stimulate the DQ2-Glia-α2 specific T cell clone (**Fig. 1C**).

Based on our observation that the α-gliadins expressed from locus *Gli-A2* of the T. *aestivum* A genome carry a variant DQ2-Glia-α2 epitope in which the proline at p8 has been replaced by a serine, and our experimental result that introducing this substitution in a peptide leads to loss of DQ2-Glia-α2 T cell stimulatory properties (**Table 6**), we wanted to determine if this amino acid substitution was indeed the cause of loss of immunogenicity. For this purpose we sequenced the α-gliadin locus of three diploid wheat (*T. monococcum*, A genome) accessions. In agreement with the results from hexaploid wheat transcripts (**Fig. S2**) we observed that the α-gliadin transcripts from *T. monococcum* contain only a single form of DQ2-Glia-α1 and DQ2-Glia-α2. Moreover, in the DQ2-Glia-α2 epitope the proline at p8 was consistently replaced by a serine (**Table S1**). Together these results establish that this naturally occurring single amino acid substitution is sufficient to completely eliminate the T cell stimulatory properties of the DQ2-Glia-α2 epitope in gluten.

Differential reactivity of the DQ2-Glia-α3 specific T cells towards the extracts of the diploids correlated with an arginine to proline replacement at p2 in α-gliadins derived from the S-genome, FRPQQPYPQ→ FPPQQPYPQ (**Table 6**).

### Elimination of α-gliadin toxicity by a naturally occurring single amino acid substitution

**Figure 2:**
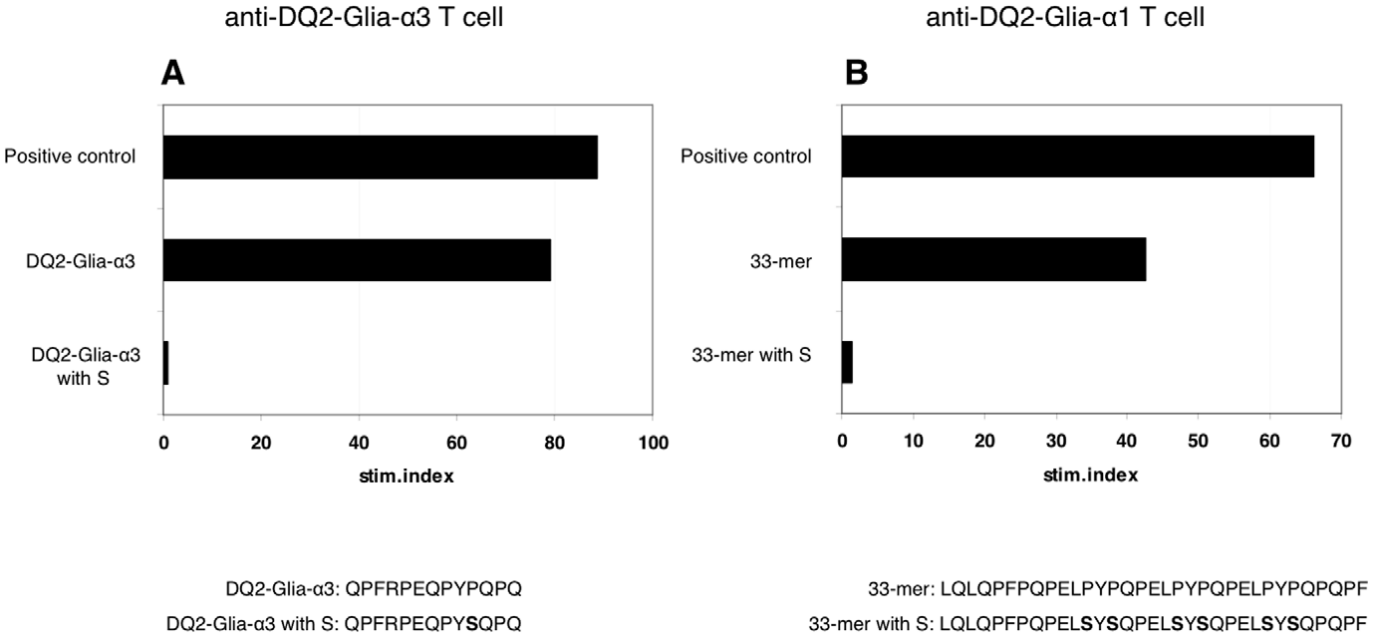
Amino acid substitution eliminates toxicity of known α-gliadin epitopes. The DQ2-Glia-α3 epitope and the known 33-mer were synthesized in deamidated form either as the original sequence or after substitution in each epitope of the prolines at position 8 with serine. These peptides were tested in T cell proliferation assays. A: T cell proliferation assay using a DQ2-Glia-α3 T cell clone. B: T cell proliferation assay using a DQ2-Glia-α1 T cell clone. Positive control: synthetic peptide encoding the specific minimal T cell epitope. Stim. index: stimulation index defined as the specific proliferation of a sample divided by the background proliferation.

The large majority of known antigenic peptides derived from wheat gluten, as well as homologous peptides derived from the hordeins from barley and the secalins from rye, contain a proline at p8 [24]. Based on our observation that a single proline to serine substitution at p8 induced unresponsiveness of DQ2-Glia-α2 specific T cells and the previous observation that a similar substitution at p8 of the DQ2-Glia-α1 epitope induced T cell unresponsiveness (**Table 6**; peptide no. 4), we investigated if a similar substitution would also eliminate the antigenic properties of the DQ2-Glia-α3 epitope. Wild type versions of the DQ2-Glia-α1, -α2 and -α3 epitopes as well as versions in which the proline at p8 was substituted by a serine were synthesized and tested in T cell proliferation studies. As expected neither the substituted DQ2-Glia-α1 epitope (QLQPFPQPEL**S**YPQPQ) nor the substituted DQ2-Glia-α2 epitope (QLQPFPQPELPY**S**QPQ) induced T cell proliferation of respectively DQ2-Glia-α1 and DQ2-Glia-α2 specific T cells (**Table 6** **)**. Likewise, the substituted DQ2-Glia-α3 epitope failed to induce T cell activation (**Fig. 2A**).

We also analysed the effects of proline to serine substitutions in the 33-mer α-gliadin derived peptide that encodes 6 partially overlapping antigenic DQ2-Glia-α1 and -α2 sequences **(** **Fig. 2B** **)** as well as in an elongated version of the 33-mer which also encodes the DQ2-Glia-α3 epitope **(Table S2)**. In all cases, the proline to serine substitutions completely abrogated the response of DQ2-Glia-α1, DQ2-Glia-α2 and DQ2-Glia-α3 specific T cell clones (**Fig. 2B** **, Table S2**).

**Figure 3:**
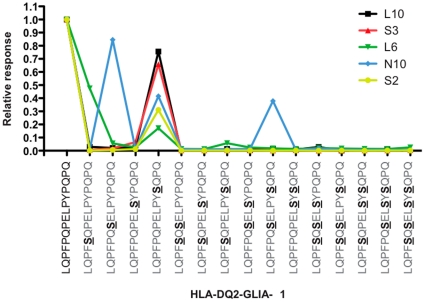
Response of DQ2-Glia-α1 epitope specific T cell clones against single and multiple P to S substituted peptides. Five T cell clones derived from 3 CD patients were tested against the deamidated form of the DQ2-Glia-α1 peptide (sequence PFPQPELPY) and variants thereof in which prolines at position 3, 5, 8 and 10 were systematically substituted for serine, both as single substitutions and in all possible combinations. Shown is the response to the substituted peptides relative to unsubstituted DQ2-Glia-α1 epitope. The introduced substitutions are underlined. The most C-terminal substituted proline at position 10 lies outside the 9 amino acid core of the T cell stimulatory peptide.

Finally, we tested the effect of systematic introduction of single, double, triple and quadruple P to S substitutions in the DQ2-Glia-α-1 peptide with five T cell clones isolated from small intestinal biopsies of 3 CD patients. T cell responses of single T cell clones were only observed for two of the single P to S substitutions at p3 and p5. In contrast, with a single P to S substitution at p8 and in all but one of the other cases the substitutions completely eliminated the T cell response of all five T cell clones (**Fig. 3**)

Thus, the naturally occurring proline to serine substitution constitutes a universal approach to remove the antigenic properties of HLA-DQ2 restricted α-gliadin peptides.

## Discussion

Although T cell responses to peptides derived from α-, γ- and ω-gliadins as well as from HMW- and LMW-glutenins have been described, various studies have indicated that the α-gliadins are among the most immunogenic regarding CD [8]–[10], [13], [25]. A crucial step towards the elimination of gluten toxicity would thus be the elimination of T cell stimulatory α-gliadin sequences. Our extensive genetic analysis of over 3000 α-gliadin transcripts from different bread wheat accessions showed a high heterogeneity of the α-gliadins genes and considerable differences in the number of T cell stimulatory sequences encoded by the various α-gliadin genes. We identified three major factors determining these differences: i) the length of tandem repeats of antigenic sequences; ii) natural amino acid substitutions that affect the antigenicity of T cell epitopes, and iii) amino acid deletions that eliminate the antigenicity of T cell epitopes.

The α-gliadins from locus *Gli-D2* generally encode several copies of both the DQ2-Glia-α1 and DQ2-Glia-α2 epitopes in addition to the DQ2-Glia-α3 and DQ8-Glia-α1 epitopes, *Gli-A2* genes usually encode only the DQ2-Glia-α1 and DQ2-Glia-α3 epitopes while the *Gli-B2* genes encode no or at most one DQ2-Glia-α1 epitope, next to the DQ8-Glia-α1 epitopes. The 33-mer sequence with 6 T cell stimulatory sequences [13] was only found in a minority of the α-gliadins analyzed, all of which are expressed from *Gli-D2*. Many natural occuring amino acid substitutions affecting the antigenicity of the canonical α-gliadin peptides were identified. Typical examples are the proline to serine substitution at p8 in the DQ2-Glia-α2 epitope and the arginine to proline substitution at p2 in the DQ2-Glia-α3 epitope, that both completely eliminate the T cell stimulatory properties of these peptides.

The analysis of gluten extracts from the diploid wheat varieties underscores these observations and provides a molecular basis for the previous observation that A-genome diploid *T. monococcum* varieties lack the DQ2-Glia-α2 epitope [18]. All α-gliadins from this genome encode an altered version of the DQ2-Glia-α2 epitope with a serine at p8 that fails to induce T cell responses. We observed that a similar substitution eliminates the T cell stimulatory properties of the DQ2-Glia-α1 and DQ2-Glia-α3 epitopes. The more variable reaction pattern of T-cells and antibodies towards gluten extracts of the S-genomes reflects the higher level of genetic variation in these outcrossing species. These α-gliadins contain another variant of the DQ2-Glia-α1 and DQ2-Glia-α2 region that does not incude the A-genome specific serine at p8. Furthermore, amino acid deletions in the canonical α-gliadin peptides prohibit binding to HLA-DQ2 and hence T cell recognition. A typical example is the deletion of the glutamine at p4 in the DQ2-Glia-α1 epitope which generates a peptide that no longer binds to HLA-DQ2, presumably due to defective docking of the anchor residues into their respective pockets in the HLA-DQ2 molecule. Our results confirm previous observations [18], [19] that the α-gliadin locus *Gli-D2* encodes the most toxic α-gliadins while substantially less toxicity is associated with those from *Gli-A2* and *Gli-B2*, but also provide the molecular basis for these differences.

Unfortunately, due to the complexity of both the *Gli-2* gene family and the wheat genome, it will be a difficult task to generate tetraploid pasta and hexaploid bread wheat that is entirely safe for consumption by all CD patients by conventional breeding methods. Our results now provide a rationale for an alternative approach as we demonstrate that by the introduction of naturally occurring amino acid substitutions the toxicity of all four T cell epitopes in α-gliadins can be eliminated. Using novel methods such as zinc finger nucleases [26]–[28] we can introduce the underlying SNPs as specific mutations into the α-gliadin genes of wheat to eliminate toxicity completely. Technically this will not be easy, but our results indicate precisely the three complementary sets of actions that need to be performed.

i) In the *Gli-B2*-derived α-gliadins analyzed, none of the DQ2 epitopes are present due to a single amino acid deletion in the region encoding the DQ2-Glia-α1 and DQ2-Glia-α2 epitopes, which generates a peptide that has decreased binding affinity for HLA-DQ2 and is no longer recognized by T cells. Therefore, a single amino acid substitution in the DQ2-Glia-α3 epitope will result in a peptide that has completely lost HLA-DQ2 binding properties and T cell stimulatory properties. To eliminate the remaining DQ8-Glia-α1 epitope in some *Gli-B2* α-gliadins, a single glutamine to arginine substitution, which naturally occurs in the α-gliadins from the A-genome, would suffice. Such a minimally genetically modified B-genome α-gliadin gene would thus no longer encode any T cell stimulatory peptides. Moreover, by starting with an α-gliadin gene in which the sequence of the p31-43 peptide is naturally altered (for example the gene encoding protein no. 9 in SI 2), the chance of innate immune stimulation by a protein derived from such a gene would also be minimized.

ii) For *Gli-A2* α-gliadins, the approach to eliminate toxicity would be to introduce two proline to serine substitutions at p8 in the DQ2-Glia-α1 and DQ2-Glia-α3 epitopes present. As these α-gliadins genes encode a shorter version of the immunodominant 33-mer in which the DQ2-Glia-α-2 epitope is already non-functional, and contain a version of the DQ8-Glia-α1 epitope that has no T cell stimulatory properties, these two substitutions would completely remove toxicity in proteins encoded by such modified genes.

iii) Regarding the *Gli-D2* α-gliadins, we found that the proline to serine substitutions at p8 completely abrogated the in vitro T cell stimulatory properties of the 33-mer peptide and of an elongated version of the 33-mer that also encodes the DQ2-Glia-α3 epitope. This result underscores our previous observation that most T cell stimulatory gluten peptides have a proline at p8 [24]. However, to render α-gliadins from the D-genome non-toxic, up to seven substitutions need to be introduced in a single gene.

An alternative approach is to design safe α-gliadin genes that can subsequently be introduced into celiac disease safe cereals such as rice or maize, for the production of gluten proteins. As such modified proteins will be very similar to existing α-gliadins they will most likely have indistinguishable technological properties. Thus, such gluten proteins could enhance the baking properties of these cereal crops, or they could be extracted from these crops and used as an ingredient to generate novel high quality foods for celiac disease patients. For the generation of high quality cereals that can replace wheat-based products the simple introduction of detoxified α-gliadins is unlikely to be sufficient, as baking quality is mostly determined by the HMW and LMW glutenin proteins. Therefore, additional studies will have to investigate how other gluten proteins can be detoxified as there is substantial evidence that these contain T cell stimulatory peptides as well. In previous studies we provided evidence that such epitopes can be found in the γ-gliadins as well as in the LMW- and HMW-glutenins [11], [12] and others have extended these observations [14], [25]. In particular, we found that T cell responses to LMW-glutenins were found in children while these are much less frequent in adults [12], [25]. Moreover, in a recent study a highly antigenic ω-gliadin peptide was described [25] that is identical to an antigenic hordein-derived peptide reported by us earlier [20]. In essence this hordein/omega peptide is a sequence variant of the DQ2-Glia-α1 peptide and also carries a proline at the p8 position [20]. It is therefore feasible that the toxicity of this peptide can be eliminated by a proline to serine substitution at p8 as well. Preliminary results show that amino acid substitutions similar to those that destroy the T cell stimulatory properties in α-gliadins might also be effective for γ-gliadin derived epitopes (Salentijn et al, in prep), but it is likely that for the LMW- and HMW-glutenins other approaches will be required. This will be the subject of further studies.

In conclusion, we have demonstrated that by utilizing naturally occurring amino acid substitutions the toxicity of the four T cell epitopes in α-gliadins can be eliminated. Such modified proteins will most likely display indistinguishable technological properties. Thus, our results provide a rational approach to eliminate CD related toxicity from α-gliadins, which represents a first but crucial step towards the realization of safe gluten containing food products for CD patients.

## Materials and Methods

### Analysis of α-gliadin transcripts from diploid wheat varieties

*T. monococcum* accessions CGN10500, CGN12035 and CGN10555 (CGN, Wageningen, The Netherlands) were used for cloning and sequencing of α-gliadin transcripts. Plants were grown under greenhouse conditions. Developing green kernels of single plants were harvested and used for RNA isolation according to Doyle and Doyle [29] but with 1% (w/v) poly-(vinylpyrrolidone)-10 in the extraction buffer. For the production of first strand cDNA 1 µg of total RNA was treated with DNAse I (Invitrogen, amplification grade; 18068-015) followed by RT PCR (Invitrogen SuperScript III First-Strand Synthesis System for RT-PCR; 18080-051) using random hexamer primers in a final reaction volume of 20 µl. Primers specific for α-gliadin genes, located on the conserved sequences at the 5′ and 3′ end of the coding region of the α-gliadin, were used to amplify α-gliadin transcripts from the cDNA samples (αF1: 5′-atgaaRaCmtttcYcatc and α5R: 5′-gttagtaccgaNgatgcc). The PCR conditions: 5 min. at 94°C, 30 cycles (94°C for 1 min., 49°C for 1 min. and 72°C for 2 min), 72°C for 10 min, 25 µl reaction volume. The PCR products were cloned and sequenced.

### Characterization of expressed α-gliadin sequences

Over 3200 *T. aestivum* expressed sequence tags (ESTs) and mRNAs designated as α-gliadin or α/β-gliadin were downloaded from the NCBI UniGene library (Ta.15268, Ta.23792, Ta.24084, Ta.25210, Ta.27702, Ta.28482) (http://www.ncbi.nml.nih.gov/UniGene) on 13 April 2007. The sequences were from *T. aestivum* libraries of various tissues, treatments and cultivars (Chinese Spring 34.2%; Glenlea 20.8%; Cheyenne 10.4%; Recital 7.8%; Mercia 7.2%; unknown cultivar 8.9%; Wyuna 3.2%; HiLine 2.9%; Butte 86, 2.4%; Hartog 1.5%; Soleil 0.6%; Nostar 0.1%; Novobirskaya 67, 0.1%). The DNA sequences were aligned using the SeqMan II (DNAstar) and first assembled at a minimum match percentage of 60%, gap lengths 3200, maximum match size 50 bp. BLAST analysis of the contigs was performed to verify the α-gliadin identity of the contigs and short (<100 bp) and bad sequences were discarded. The transcript contigs of ≥60% homologous sequences were trimmed up to the start and stop codons. Next, the sequences were reassembled at 90% homology, which resulted in 55 α-gliadin transcript contigs containing 1 to 475 sequences. The 3′ end was covered by 50 contigs (2911 transcripts) whereas the 5′ end was present in 30 contigs (2753 transcripts). The consensus of these contigs were saved in separate files and used for phylogenetic studies to deduce the genome of origin of the sequences in each contig.

### Phylogenic analysis

With the aim to deduce the locus, *Gli-A2*, *Gli-B2* or *Gli-D2,* from which the transcripts were expressed, the 55 α-gliadin EST consensus nucleotide sequences obtained from clustering, were aligned using Clustal W, *MEGA* 4 [30], together with 56 genomic DNA sequences of known origin, i.e. derived from the diploid wheat species *T. monococcum* (A genome), *T. speltoides* (S genome) and *Aegilops tauschii* (D genome) [18] and DNA sequences that were previously assigned to a locus [31]. The sequences that covered the 5′ region of the α-gliadin sequences were trimmed up to the start and up to nucleotides coding for the conserved amino acid motif PIS, located just in front of the first glutamine repeat, to cover the same region and subsequently used to generate a Neighbor-Joining tree (bootstrap test of 1000 replicates, pairwise deletion of gaps and missing data, Kimura 2-parameter, Substitutions to Include Transitions + Transversions; Pattern among Lineages Homogeneous, Uniform rates among sites, number of sites = 750, in MEGA 4)(Fig. S1).

### Sequence variation in epitope regions

To analyze all sequence variation, the 55 α-gliadin EST contigs, now assigned to a specific chromosome, were reassembled one by one at 99–100% match (SeqMan II, Lasergene, DNAstar). This yielded 717 different allelic variants. The consensus nucleotide sequences were translated (MEGA 4) and explored for epitopes and surrounding sequence regions using a text explorer program (PatternResearch, in house developed) after which the output file was analysed in Excel.

### T cell clones, T cell proliferation and HLA-DQ2 binding assays

Gluten specific T cell clones were generated from small intestinal biopsies of celiac disease patients as described before [8], [11], [12]. All patients signed an informed consent form which was approved by the hospital ethics committee. Proliferation assays were performed in triplicate in 150 µl Iscove's Modified Dulbecco's Medium (Bio Whittaker, Verviers, Belgium) with 10% pooled normal human serum in 96 well flat-bottom plates using 2×104 gluten specific T cells stimulated with 105 irradiated HLA-DQ2-matched allogeneic peripheral blood mononuclear cells (3000 rad) in the presence or absence of antigen (1–10 µg/ml) [8], [11], [12]. After 2 days ^3^H-thymidine (0.5 µCi/well) was added to the cultures, and 18–20 hours thereafter the cells were harvested. ^3^H-thymidine incorporation in the T cell DNA was determined with a liquid scintillation counter (1205 Betaplate Liquid Scintillation Counter, LKB Instruments, Gaithersburg, MD)**.** A binding assay was performed as described previously [24].

## Supporting Information

Figure S1 **Phylogenetic analysis of α-gliadin sequences.** A neighbor-joining tree was made with 55 EST consensus nucleotide sequences from hexaploid bread wheat together with 56 genomic DNA sequences derived from the diploid wheat species *T. monococcum* (A genome, green dots), *T. speltoides* (S/B genome, blue dots) and *Aegilops tauschii* (D genome, red dots), after alignment using Clustal W. The EST sequences (black dots) can be assigned to their locus in hexaploid bread wheat as they cluster into the same three groups as the sequences from the diploid species (A genome  =  locus *Gli-A2*, S/B genome  =  locus *Gli-B2*, D genome  =  locus *Gli-D2* [17]).
(TIF)

Figure S2 **Sequence variation in the N-terminal and C-terminal regions of α-gliadin genes from hexaploid wheat.** The 23 most frequently found expressed sequence tag (EST) contigs were translated, the amino acid sequences were aligned and grouped per chromosomal location (the *Gli-A2* locus on chromosome 6AS, *Gli-B2* on 6BS, and *Gli-D2* on 6DS) to present the variation in various gluten epitope regions. Note the large differences in the number of times (N) each sequence was present in the set of ESTs. In red: amino acid variation in the sequence. In back: chymotrypsin or trypsin sites (>72% affinity).
(TIF)

Figure S3 **DQ2-Glia-α1 variants.** The effect of various amino acid substitutions on the ability of the DQ2-Glia-α1 peptide to stimulate DQ2-Glia-α1 T cells.
(TIF)

Table S1 **Amino acid sequences of the DQ2-Glia-α1/-α2/-α3 region of α-gliadin cDNA transcripts from three diploid** ***T.monococcum*** **accessions (diploid, AA genome).** The gene sequences have been submitted as accession numbers HQ317881-HQ317890.
(DOC)

Table S2 **The effects of proline to serine substitutions in an elongated version of the 33-mer, which also encodes the DQ2-Glia-α3 epitope.**
(DOC)
