# Correction: A Universal Approach to Eliminate Antigenic Properties of Alpha-Gliadin Peptides in Celiac Disease

**DOI:** 10.1371/annotation/cdf9d655-07e8-4081-ad86-a00c89fa001a

**Published:** 2012-09-19

**Authors:** Cristina Mitea, Elma M. J. Salentijn, Peter van Veelen, Svetlana V. Goryunova, Ingrid M. van der Meer, Hetty C. van den Broeck, Jorge R. Mujico, Veronica Montserrat, Luud J. W. J. Gilissen, Jan Wouter Drijfhout, Liesbeth Dekking, Frits Koning, Marinus J. M. Smulders

The eighth author's name was spelled incorrectly. The correct name is: Veronica Montserrat.

