# Supplementary figures and images for: A Universal Approach to Eliminate Antigenic Properties of Alpha-Gliadin Peptides in Celiac Disease
Source: PLoS One. 2010 Dec 16;5(12):e15637. doi: 10.1371/journal.pone.0015637 (PMC3002971; doi:10.1371/journal.pone.0015637)

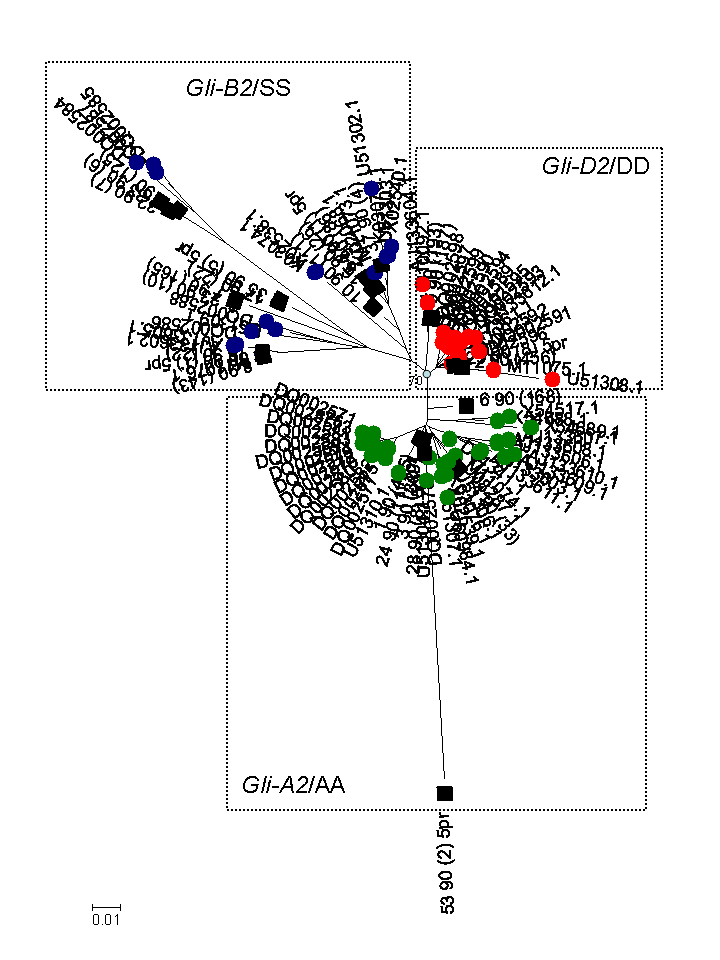

Supplement: Figure S1 — Phylogenetic analysis of α-gliadin sequences. A neighbor-joining tree was made with 55 EST consensus nucleotide sequences from hexaploid bread wheat together with 56 genomic DNA sequences derived from the diploid wheat species T. monococcum (A genome, green dots), T. speltoides (S/B genome, blue dots) and Aegilops tauschii (D genome, red dots), after alignment using Clustal W. The EST sequences (black dots) can be assigned to their locus in hexaploid bread wheat as they cluster into the same three groups as the sequences from the diploid species (A genome = locus Gli-A2, S/B genome = locus Gli-B2, D genome = locus Gli-D2 [17]). (TIF) [file pone.0015637.s001.tif]

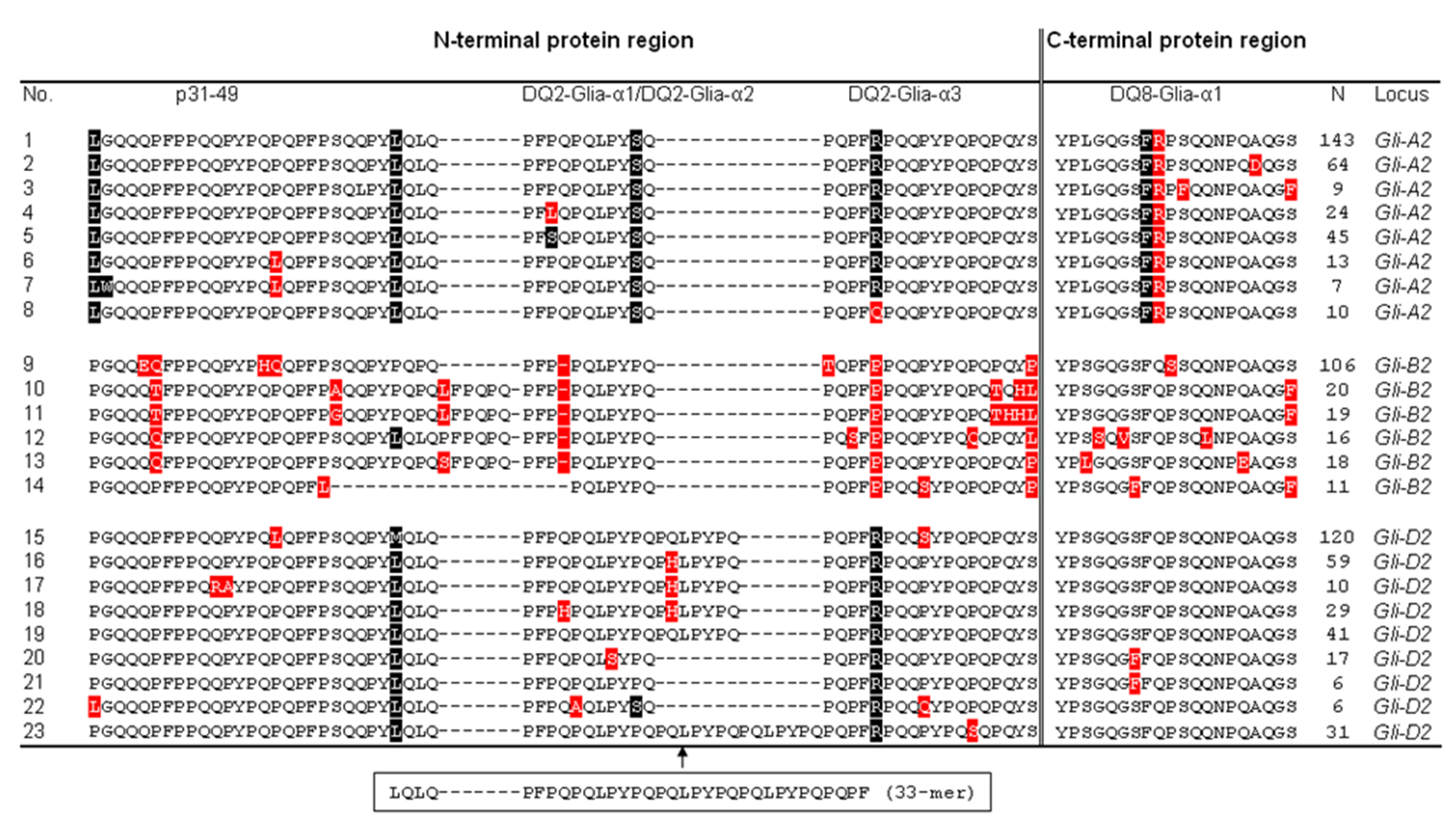

Supplement: Figure S2 — Sequence variation in the N-terminal and C-terminal regions of α-gliadin genes from hexaploid wheat. The 23 most frequently found expressed sequence tag (EST) contigs were translated, the amino acid sequences were aligned and grouped per chromosomal location (the Gli-A2 locus on chromosome 6AS, Gli-B2 on 6BS, and Gli-D2 on 6DS) to present the variation in various gluten epitope regions. Note the large differences in the number of times (N) each sequence was present in the set of ESTs. In red: amino acid variation in the sequence. In back: chymotrypsin or trypsin sites (>72% affinity). (TIF) [file pone.0015637.s002.tif]

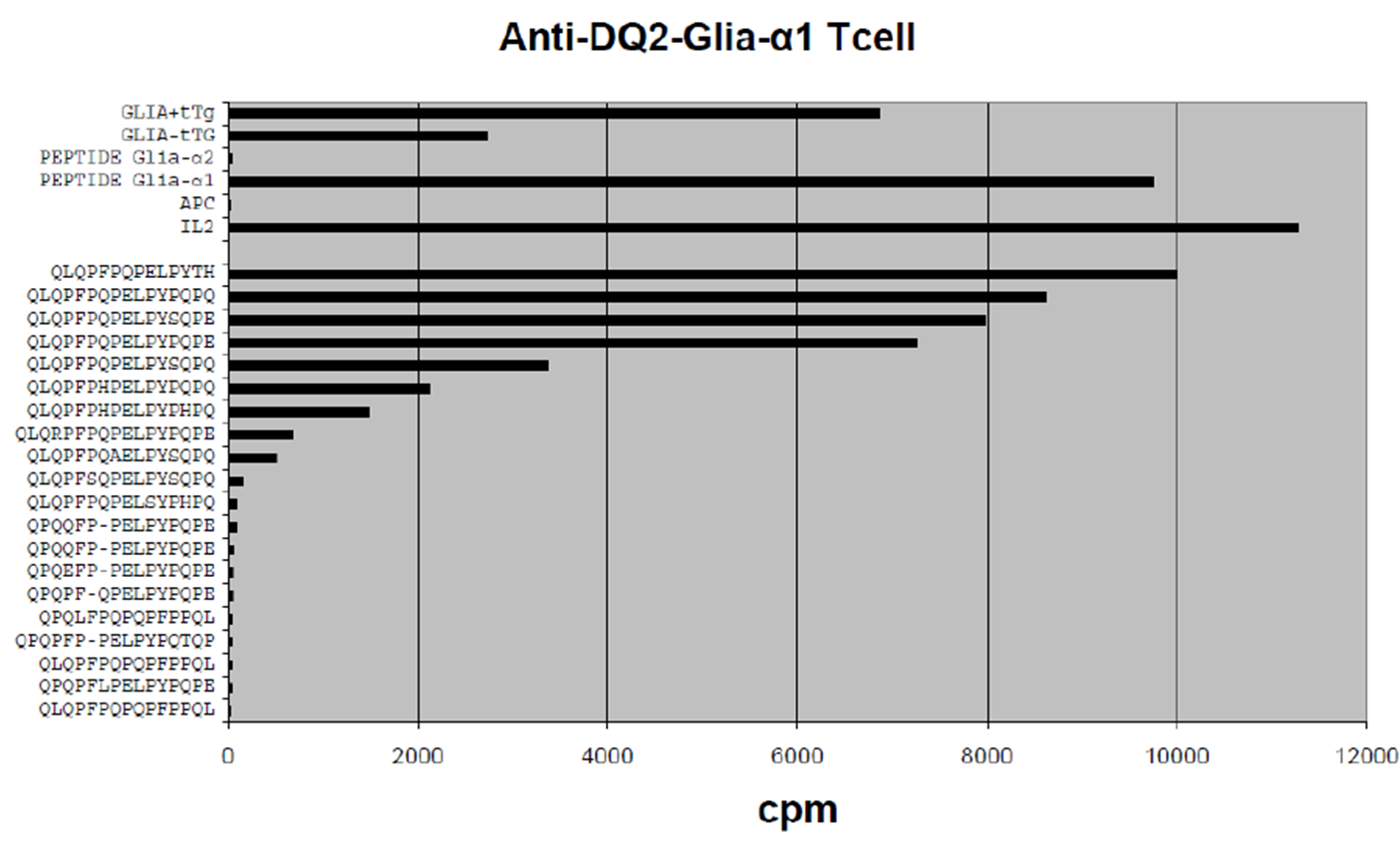

Supplement: Figure S3 — DQ2-Glia-α1 variants. The effect of various amino acid substitutions on the ability of the DQ2-Glia-α1 peptide to stimulate DQ2-Glia-α1 T cells. (TIF) [file pone.0015637.s003.tif]
