# Supplementary material for: A Universal Approach to Eliminate Antigenic Properties of Alpha-Gliadin Peptides in Celiac Disease
Source: PLoS One. 2010 Dec 16;5(12):e15637. doi: 10.1371/journal.pone.0015637 (PMC3002971; doi:10.1371/journal.pone.0015637)
Supplement: Table S1 — Amino acid sequences of the DQ2-Glia-α1/-α2/-α3 region of α-gliadin cDNA transcripts from three diploid T.monococcum accessions (diploid, AA genome). The gene sequences have been submitted as accession numbers HQ317881-HQ317890. (DOC) [file pone.0015637.s004.doc]

| *T.monococcum* accession | Ntotal | DQ2-Glia-α1/-α2/-α3 region | % |
| --- | --- | --- | --- |
| CGN10500 | 64 | PQLQPFPSQQPYLQLQPFPQPQLPY**S**QPQPFRPQQPYPQPQPQYS | 45% (n=29) |
|  |  | PQ**P**QPFPSQQPYLQLQPFPQPQLPY**S**QPQPFRPQQPYPQPQPQYS | 55% (n=35) |
|  |  |  |  |
| CGN12035 | 8 | PQLQPFPSQQPYLQLQPFPQPQLPY**S**QPQPFRPQQPYPQPQPQYS | 100% (n=8) |
|  |  |  |  |
| CGN10555 | 39 | PQLQPFPSQQPYLQLQPFPQPQLPY**S**QPQPFRPQQPYPQPQPQYS | 62% (n=24) |
|  |  | PQ**P**QPFPSQQPYLQLQPFPQPQLPY**S**QPQPFRPQQPYPQPQPQYS | 38% (n=15) |
