# Supplementary material for: A Universal Approach to Eliminate Antigenic Properties of Alpha-Gliadin Peptides in Celiac Disease
Source: PLoS One. 2010 Dec 16;5(12):e15637. doi: 10.1371/journal.pone.0015637 (PMC3002971; doi:10.1371/journal.pone.0015637)
Supplement: Table S2 — The effects of proline to serine substitutions in an elongated version of the 33-mer, which also encodes the DQ2-Glia-α3 epitope. (DOC) [file pone.0015637.s005.doc]

| Stimulator peptide | SI |
| --- | --- |
| DQ2-Glia-α3 eitope | 59 |
| DQ2-Glia-α3 substituted epitope | 1 |
| Elongated 33-mer epitope | 49 |
| Elongated substituted 33-mer epitope | 1 |

Table S2
